# Spatiotemporal dynamics of emerging pathogens in questing *Ixodes ricinus*

**DOI:** 10.3389/fcimb.2013.00036

**Published:** 2013-07-30

**Authors:** Elena Claudia Coipan, Setareh Jahfari, Manoj Fonville, Catharina B. Maassen, Joke van der Giessen, Willem Takken, Katsuhisa Takumi, Hein Sprong

**Affiliations:** ^1^Centre for Infectious Disease Control, National Institute for Public Health and Environment (RIVM)Bilthoven, Netherlands; ^2^Laboratory of Entomology, Wageningen UniversityWageningen, Netherlands; ^3^Food Chain Quality, Antibiotics and Zoonoses Research Group, Central Veterinary InstituteLelystad, Netherlands

**Keywords:** vector-borne disease, *Borrelia burgdorferi*, *Candidatus Neoehrlichia mikurensis*, *Rickettsia helvetica*, *Rickettsia conorii*, *Anaplasma phagocytophilum*, *Babesia*, *Ixodes ricinus*

## Abstract

*Ixodes ricinus* transmits *Borrelia burgdorferi* sensu lato, the etiological agent of Lyme disease. Previous studies have also detected *Rickettsia helvetica*, *Anaplasma phagocytophilum*, *Neoehrlichia mikurensis*, and several *Babesia* species in questing ticks in The Netherlands. In this study, we assessed the acarological risk of exposure to several tick-borne pathogens (TBPs), in The Netherlands. Questing ticks were collected monthly between 2006 and 2010 at 21 sites and between 2000 and 2009 at one other site. Nymphs and adults were analysed individually for the presence of TBPs using an array-approach. Collated data of this and previous studies were used to generate, for each pathogen, a presence/absence map and to further analyse their spatiotemporal variation. *R. helvetica* (31.1%) and *B. burgdorferi* sensu lato (11.8%) had the highest overall prevalence and were detected in all areas. *N. mikurensis* (5.6%), *A. phagocytophilum* (0.8%), and *Babesia spp*. (1.7%) were detected in most, but not all areas. The prevalences of pathogens varied among the study areas from 0 to 64%, while the density of questing ticks varied from 1 to 179/100 m^2^. Overall, 37% of the ticks were infected with at least one pathogen and 6.3% with more than one pathogen. One-third of the *Borrelia*-positive ticks were infected with at least one other pathogen. Coinfection of *B. afzelii* with *N. mikurensis* and with *Babesia spp*. occurred significantly more often than single infections, indicating the existence of mutual reservoir hosts. Alternatively, coinfection of *R. helvetica* with either *B. afzelii* or *N. mikurensis* occurred significantly less frequent. The diversity of TBPs detected in *I. ricinus* in this study and the frequency of their coinfections with *B. burgdorferi* s.l., underline the need to consider them when evaluating the risks of infection and subsequently the risk of disease following a tick bite.

## Introduction

In The Netherlands, the hard tick *Ixodes ricinus* is the main vector of a variety of human pathogens. The most prevalent tick-borne disease is Lyme borreliosis (Stanek et al., 2012). This multi-systemic disorder is caused by several members of the *Borrelia burgdorferi* sensu lato complex. Of the 18 genospecies of this complex (Margos et al., 2011), *B. afzelii, B. garinii, B. spielmanii, B. bavariensis*, and *B. burgdorferi* sensu stricto have already been detected in The Netherlands, in both patients and questing ticks. *B. lusitaniae*, and *B. valaisiana* were detected in questing *I. ricinus*, but their public health significance is less clear (Collares-Pereira et al., 2004; Diza et al., 2004; De Carvalho et al., 2008; Coipan et al., 2013).

Over the last decades, the incidence of Lyme borreliosis has increased significantly in Europe (Smith and Takkinen, 2006). A long-term retrospective study among general practitioners in The Netherlands has shown a continuing increase in consultations for tick bites and *erythema migrans* in the last decade (Hofhuis et al., 2006). The incidence of *erythema migrans* patients increased from 39 per 100,000 inhabitants in 1994 to 134 per 100,000 inhabitants in 2009.

Previous studies in The Netherlands have identified the presence of other pathogens in questing *I. ricinus* as well. Human babesiosis is caused by the intraerythrocytic protozoa *Babesia divergens, B. venatorum (EU1)*, and *B. microti* (Vannier and Krause, 2012). A recent study reported these three *Babesia* species in approximately 1% of questing *I. ricinus* (Wielinga et al., 2009). The spotted fever syndrome is caused by at least 15 different *Rickettsia* species, some of which are transmitted by *I. ricinus* (Heyman et al., 2010). *Rickettsia conorii* and *R. monacensis* are probably the most common tick-borne *Rickettsiae* to cause disease in Europe (Heyman et al., 2010), whereas the pathogenicity of *R. helvetica* is still questionable (Svendsen, 2011). All three rickettsial species have been previously found in The Netherlands (Sprong et al., 2009) with local prevalences varying from <1% (*R. conorii*) to as high as 66% (*R. helvetica*). *Anaplasma phagocytophilum*, the etiologic agent of human granulocytotropic anaplasmosis (Sprong et al., 2009), has been detected in Dutch ticks in several studies (Tijsse-Klasen et al., 2010, 2011b). *Neoehrlichia mikurensis* can be considered an emerging zoonosis, as more than eight human cases have been described in Europe since 2010, while previously it was considered non-pathogenic. Despite an overall prevalence of *N. mikurensis* in questing ticks of approximately 7% (Jahfari et al., 2012), no human cases have been reported in The Netherlands.

Autochthonous tick-borne diseases other than Lyme disease have not been reported, except for a single case of granulocytotropic anaplasmosis in 1999 (Van Dobbenburgh et al., 1999). This may be caused by lower pathogenicity, lack of overt symptoms, or lack of awareness of public and health professionals.

Multiple studies have reported coinfection with some of the tick-borne pathogens (TBPs) (Belongia, 2002; Ginsberg, 2008; Nieto and Foley, 2009; Reye et al., 2010; Burri et al., 2011; Lommano et al., 2012). It is known that the severity of Lyme disease may be affected by simultaneous infections with other TBPs (Belongia, 2002; Swanson et al., 2006). Some of them, such as *A. phagocytophilum*, modulate host immunity and increase susceptibility to various second pathogens, including *B. burgdorferi* (Thomas et al., 2001; Holden et al., 2005). Thus, coinfection might be partly responsible for the variability in clinical manifestations that are usually associated with Lyme borreliosis.

The acquirement of a tick-borne disease depends on many environmental, societal, and immunological factors, but it is always preceded by the bite of a tick infected with the causal agent. Previous studies have shown that the risk of infection of humans by TBPs depends mainly on the density of questing infected ticks—the acarological risk (Glass et al., 1994, 1995; Nicholson and Mather, 1996; Dister et al., 1997; Kitron and Kazmierczak, 1997). The study of mixed infections in questing ticks might, therefore, reveal patterns of coinfection of *B. burgdorferi* s.l. with two or more other pathogens, allowing us to generate hypotheses on the transmission cycle of some more obscure pathogens from the dynamics of better-known ones. The aim of this study was to assess the acarological risk of exposure to TBPs in The Netherlands by comparing the abundances of questing ticks infected with *B. burgdorferi* s.l. and with other TBPs.

## Methods

### Collection of ticks and tick data

All ticks were collected on a monthly basis between 2006 and 2010 in 21 sites. In Duin&Kruidberg field sampling was conducted between 2000 and 2009. The sites were spread all over The Netherlands and they have been selected based on Lyme borreliosis incidence in humans, and the availability of volunteers. The same sites were described in some previous studies regarding ticks and TBPs in The Netherlands (Wielinga et al., 2006, 2009; Sprong et al., 2009; Gassner et al., 2011; Tijsse-Klasen et al., 2011a). Sampling of ticks was done by blanket dragging, using a 1 m^2^ cloth on a 100 m long transect. Based on morphological criteria, ticks were identified to species level, with stage and sex recorded. The density of ticks was estimated as the number of questing ticks *per* 100 m^2^. Additionally, data on the presence of ticks and TBPs in other 39 areas were collected from some previous studies that have used the same sampling and analysis methodology (Schouls et al., 1999; Tijsse-Klasen et al., 2010, 2011b; Jahfari et al., 2012).

### DNA extraction of ticks

All the collected ticks were immersed in 70% alcohol and stored at −20°C until the DNA extraction. DNA from questing ticks was extracted by alkaline lysis (Wielinga et al., 2006). Questing larvae were not taken into account as humans are generally bitten by either nymphs or adult *I. ricinus* (Hugli et al., 2009; Tijsse-Klasen et al., 2011b).

### PCR detection and identification of pathogens

The presence of the DNA of different TBPs (*Rickettsia spp., B. burgdorferi* s.l., *Ehrlichia/Anaplasma spp.*, and *Babesia spp*.) was determined by polymerase chain reaction (PCR) followed by reverse line blotting (RLB) as described before (Wielinga et al., 2006; Tijsse-Klasen et al., 2010). To minimize cross contamination and false-positive results, positive and negative controls were included in each batch tested by PCR and RLB assays. Furthermore, DNA extraction, PCR mix preparation, sample addition, and PCR analysis were performed in assigned separate labs. PCR products of some samples were sequenced by dideoxy-dye termination sequencing of both strands, and compared with sequences in GenBank (http://www.ncbi.nlm.nih.gov/), using BLAST (Altschul et al., 1990). The sequences were aligned and analysed using BioNumerics 6.6 (Applied Maths, Kortrijk, Belgium).

The prevalence of infection was calculated as the percentage of ticks infected with a certain microorganism.

### Acarological risk

To calculate the densities of questing ticks infected with each of the five pathogens' genera, we multiplied the prevalence of infection with the density of questing ticks in each of the investigated sites.

### Correlation between prevalence and tick density

For some pathogens, we noticed that the prevalence might correlate with the density of questing ticks at the sampling locations. To test this possibility we fitted a binomial model to our data, by defining the prevalence of infection as an exponential function of the tick density (*d*) at each sampling location. Knowing that the number of infected ticks (*k*) out of the total number of ticks tested (*n*), is binomially distributed with a probability (*p*), we used the function *p* = *aExp*[*bd*],0 < *a* < 1, to test an alternative model (*b* < 0) against a null model (*b* = 0) by a likelihood-ratio test. The alternative (decreasing exponential) model fitted significantly better to our data with *p*-value *P* ≤ 0.05.

### Seasonal dynamics

To test for the seasonal dynamics of infection in ticks, a binomial model for the probability of infection (*p*) was fitted to our data, in combination with the sampling days (*t*) and pooled over the sampling locations and development stages. The probability of infection was a logit function $p=\frac{e^{y}}{1+e^{y}}$ and we modeled seasonality by using the trigonometric function *y* = *a* + *b*cos(2π*t*/365) + *c*sin(2π*t*/365) to describe an oscillation with a period of 1 year and possible phase shift. For each pathogen, we tested the seasonal model against the non-seasonal model (*y* = *a*) based on a likelihood ratio test. The seasonal model fitted significantly better to our data with *p*-value *P* ≤ 0.05. All the statistical analyses were performed using Wolfram Mathematica 9.

## Results

The mean density of questing nymphs and adult ticks varied greatly between sites, from as low as 1 (at Houtvesterijen Heide) up to 179/100 m^2^ (at Duin&Kruidberg; Table 2), results that are consistent with previous Dutch studies (Wielinga et al., 2006).

### Pathogen detection and identification

A total of 5570 questing nymphs and adult *I. ricinus* from 22 different study areas were tested for the presence of TBPs by PCR amplification followed by RLB (Table 1). The recently identified *B. bavariensis* reacted consistently with our *B. garinii* probe (Margos et al., 2009), and therefore we grouped these two *Borrelia* genospecies. Five *Borrelia* genospecies were found in this study in all twenty-two study areas (Table 1), with the overall prevalence (11.8%) inscribed in the interval of average European prevalence (Rauter and Hartung, 2005), and comparable with previous studies in The Netherlands (Wielinga et al., 2006; Gassner et al., 2011). *B.afzelii* was predominant (6.7%), followed by *B. garinii/B. bavariensis* (1.5%), *B. valaisiana* (1.2%), and *B. burgdorferi* sensu stricto (0.2%). The remaining fraction of the *Borrelia* positive samples could not be further identified to the species level by RLB. Sequencing several of these samples revealed the presence of *B. spielmanii*, corroborating previous findings of this genospecies in The Netherlands (Wang et al., 1999). *B. lusitaniae*, which was recently found in The Netherlands (Tijsse-Klasen et al., 2010), was not detected in this study.

**Table 1:** **Presence of microorganisms in questing *I. ricinus* nymphs and adults**.

**Pathogen**	**Positive/tested *I. ricinus* (*n*)**	**Prevalence *I. ricinus* (%)**	**95% CL**		**Tested/%positive study areas**
			**LCL**	**UCL**	
*Borrelia burgdorferi* s.l.	628 (5308)	11.8	11.0	12.7	22/100
*–Borrelia afzelii (and B.ruski)*	355 (5308)	6.7	6.0	7.4	22/100
*–Borrelia garinii/B.bavariensis*	79 (5308)	1.5	1.2	1.9	22/77.3
*–Borrelia valaisiana*	64 (5308)	1.2	0.9	1.5	22/81.8
*–Borrelia burgdorferi* ss.	10 (5308)	0.2	0.1	0.4	22/36.4
*–*Untypeable *Borrelia*	133 (5308)	2.5	2.1	3.0	22/90.9
*Rickettsia helvetica*	1265 (4061)	31.1	29.7	32.6	19/100
*Rickettsia conorii*	3 (4061)	0.1	0.0	0.2	19/5.3
Untypeable *Rickettsia*	33 (4061)	0.8	0.6	1.1	19/68.4
*Anaplasma phagocytophilum*	44 (5343)	0.8	0.6	1.1	22/63.6
*Neoehrlichia mikurensis*	300 (5343)	5.6	5.0	6.3	22/81.8
*Ehrlichia canis*	5 (5343)	0.1	0	0.2	22/18.2
Untypeable *Ehrlichia*	99 (5343)	1.9	1.5	2.3	22/72.7
*Babesia microtii*	17 (4238)	0.4	0.2	0.6	19/31.6
*Babesia venatorum* (EU1)	41 (4238)	1.0	0.7	1.3	19/73.7
*Babesia divergens*	1 (4238)	0.0	0.0	0.1	19/5.3
Untypeable *Babesia*	12 (4238)	0.3	0.2	0.5	19/63.2

*Tick lysates were subjected to PCR followed by Reverse Line Blotting. PCR products that specifically reacted to the generic (“catch all”) probes, but that could not be further specified to the (geno)species level were designated as Untypeable. Reverse Line Blot analysis could not distinguish between B. garinii and the recently identified B. bavariensis. Calculations of prevalence were based on all tick lysates that were analysed (n)*.

*CL, confidence limits; LCL, lower confidence limit; UCL, upper confidence limit*.

*R. helvetica* was most frequently detected in tick lysates, its 31.1% average prevalence (Table 1) being among one of the highest in Europe [range 1.5 to more than 40.6% (Christova et al., 2003; Severinsson et al., 2010)]. A previous study from our laboratory found *R. helvetica* not only in vertebrate hosts, but also in tick larvae at comparable prevalences as for the other tick stages, indicating a high efficiency of transovarial transmission (Sprong et al., 2009). Thirty-three *Rickettsia* isolates could not be identified up to the species level by RLB. Sequencing of these samples revealed the presence of *R. monacensis*, which was reported in The Netherlands before (Sprong et al., 2009). *Rickettsia conorii* was detected in only three questing ticks from one study area (Veldhoven). *A. phagocytophilum*-infected ticks were recorded with an overall prevalence of only 0.8% (Table 1). *N. mikurensis* DNA was found with a global prevalence of 5.6% (Table 1). *Ehrlichia canis* DNA was detected in only 5 tick lysates from four different study areas, which resulted in an overall prevalence of 0.1% (5/5343). Ninety-nine *Ehrlichia* isolates could not be identified to the species level neither by RLB nor by sequencing. *Babesia venatorum*, formerly also known as *B.EU1* (Duh et al., 2005), was present with a global prevalence of 1.0% (41/4238). The prevalence of *B. microti* in questing ticks was 0.4% (17/4238), and the protozoan was detected in 6 from 19 sites. Only one tick from the Duin&Kruidberg area contained the DNA of previously reported *B. divergens* (Wielinga et al., 2009). Twelve *Babesia sp*. could not be further identified by neither RLB, or sequencing. The average prevalence of *Babesia*-positive ticks in the study areas was 1.6% (Table 1).

### Spatial distribution and variation

**Figure 1:**
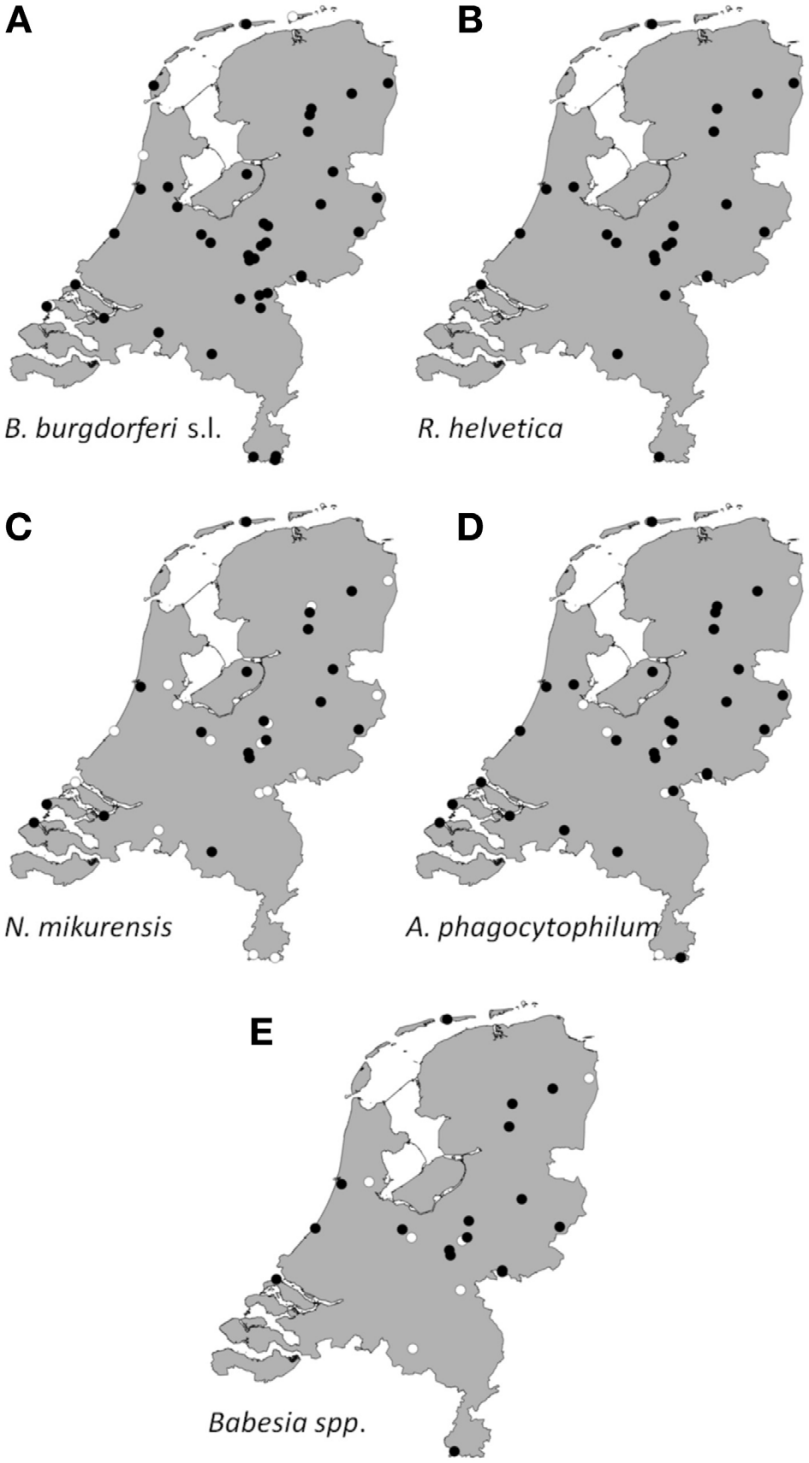
**Aggregated presence/absence map of questing *I. ricinus* nymphs/adults infected with *B. burgdorferi* s.l. **(A)**, *R. helvetica***(B)**, *N. mikurensis***(C)**, *A. phagocytophilum***(D)**, *Babesia* species **(E)**.** The black dots represent presence of the microorganism; the white ones represent absence. Presence/absence points from previous studies were also incorporated.

Collated data were used to generate presence/absence maps of the five major TBPs in The Netherlands (Figure 1). The presence/absence of *Borrelia spp., R. helvetica, A. phagocytophilum, N. mikurensis*, and *Babesia spp*. was assessed for 61, 24, 39, 39, and 25 locations, respectively. The presence of these pathogens was observed in 58, 24, 33, 20 and 18 areas, respectively, heterogeneously distributed across The Netherlands. The few absence points were scattered over The Netherlands as well, and did not cluster in any geographic region (Figure 1).

*Borrelia* prevalence was between 5% (Houtvesterijen Heide) and 50% (Bellingwedde; where only six ticks were tested), while for *R. helvetica* it varied even more, from 3% in some sites (Apeldoorn), to 64% in others (Duin&Kruidberg) (Table 2). Lower variations in prevalences were observed for *N. mikurensis*, *A. phagocytophilum* and *Babesia spp*. (Table 2). For *N. mikurensis*, the prevalence was on average of 5%, but some areas displayed values of over 10% (Table 2). *Babesia spp*. showed an overall prevalence of 1.7%, similarly to Germany and Luxembourg (Hartelt et al., 2004; Reye et al., 2010). *A. phagocytophilum* was the least prevalent pathogen in our study, with a mean prevalence of 0.8%—comparable with the 0.5–1% prevalence found in different European countries (Koci et al., 2007; Hildebrandt et al., 2010; Severinsson et al., 2010). However, one of the sites displayed a 10-fold higher prevalence than average (Bilthoven 8%, Table 2).

**Table 2:** **Prevalence (%) of the five major pathogens found in the 22 study areas**.

**Geographic location**	***B. burgdorferi* s.l.**			***R. helvetica***			***N. mikurensis***			***A. phagocytophylum***			***Babesia spp*.**			**Density (/100 m^2^)**	
	**+**	**T**	**%**	**+**	**T**	**%**	**+**	**T**	**%**	**+**	**T**	**%**	**+**	**T**	**%**	**Nymphs**	**Adults**
Apeldoorn	15	38	39	1	38	3	3	38	8	0	38	0	5	38	13	17	5
Appelscha	10	79	13	11	76	14	3	79	4	0	79	0	4	79	5	19	5
Bellingwedde	3	6	50	2	6	33	0	6	0	0	6	0	0	6	0	7	0.3
Bijlmerweide	34	330	10			ND	1	330	0.3	0	330	0			ND	12	1
Bilthoven	4	40	10	6	40	15	0	40	0	3	40	8	1	40	3	9	3
Duin& Kruidberg	123	1640	8	848	1327	64	113	1676	7	11	1676	1	12	1499	1	160	19
Ede	48	354	14	23	354	6	36	353	10	3	353	1	2	353	1	54	7
Eijsden	28	232	12	23	232	10	0	232	0	1	232	0.4	10	232	4	34	1
Gieten	10	136	7	31	136	23	6	136	4	2	136	1	2	136	1	59	5
Haaksbergen	9	105	9	11	105	10	1	105	1	4	105	4	2	105	2	77	2
Hoge Veluwe	2	8	25	2	8	25	0	8	0	0	8	0	0	8	0	25	1
Hoog Baarlo	28	311	9	24	311	8	2	311	1	4	311	1	9	311	3	34	2
Hoogeveen	47	163	29	48	163	29	11	163	7	0	163	0	5	163	3	63	3
Houtvest_Bos	49	510	10			ND	35	510	7	4	510	1			ND	32	2
Houtvest_Heide	4	88	5			ND	5	88	6	1	88	1			ND	1	0.2
Kwade Hoek	43	162	27	13	162	8	23	162	14	0	162	0	3	162	2	9	4
Montferland	18	1470	12	12	147	8	11	147	7	0	147	0	3	147	2	40	3
Nijverdal	24	127	19	13	127	10	18	127	14	1	127	1	8	127	6	34	2
Ruinen	25	94	27	30	94	32	2	94	2	2	94	2	1	94	1	18	1
Twiske	46	292	16	62	292	21	13	292	4	1	292	0.3	0	292	0	36	2
Veldhoven	25	242	10	14	239	6	13	242	5	6	242	2	1	242	0.4	47	17
Wassenaar	33	204	15	91	204	45	4	204	2	1	204	0	3	204	1	46	3
Total/Average	628	5308	11.8	1265	4061	31.1	300	5343	5.6	44	5343	0.8	71	4238	1.7	38	4

*Tick density/activity of each study area was expressed as the average density/activity of the questing ticks collected from April to September from at least three consecutive years. Average prevalences of the study areas (n = 22) were calculated. +, positive samples; T, tested; ND, Not determined*.

**Figure 2:**
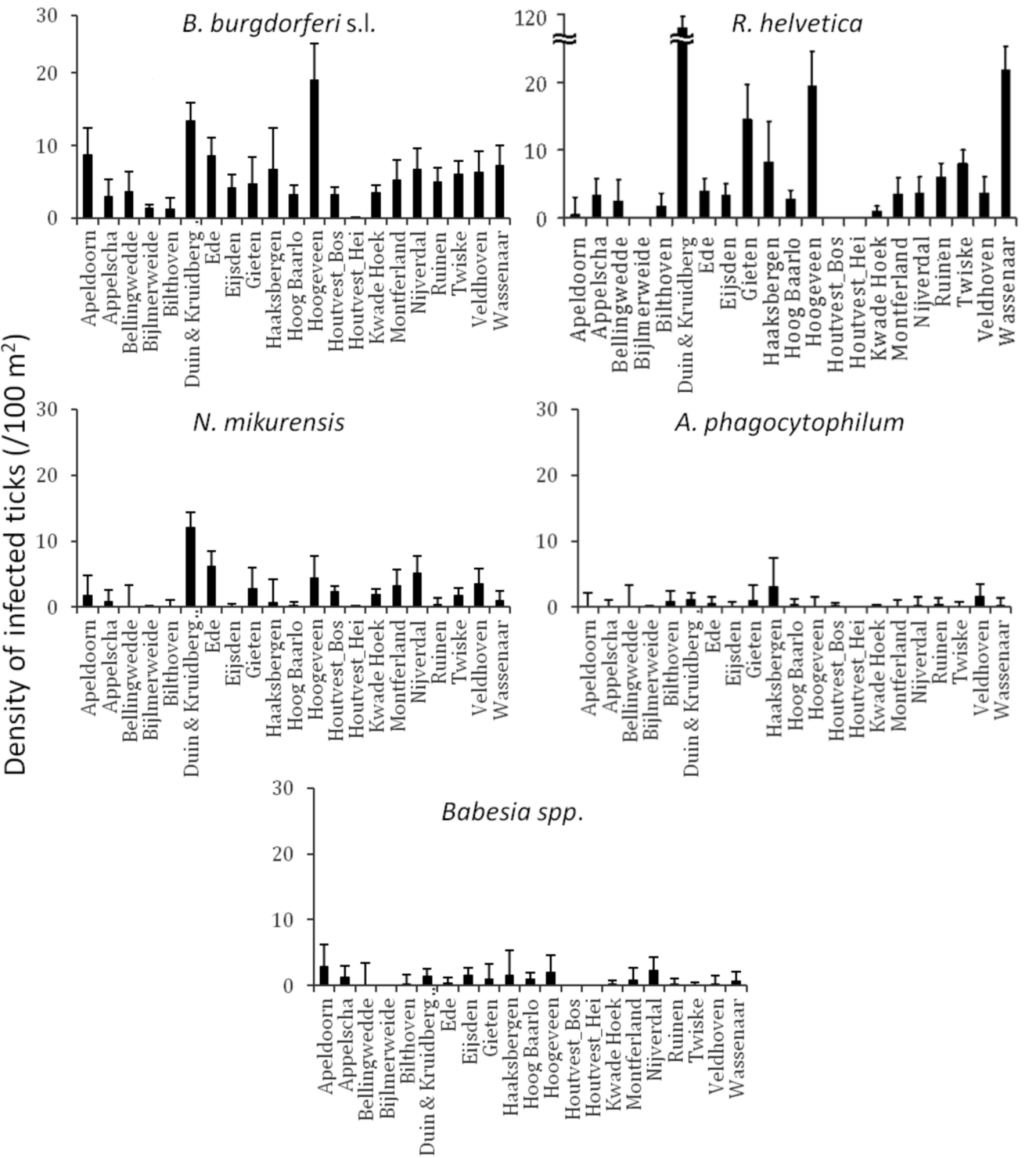
**Identification of high risk-areas depends on both prevalence and tick density/activity.** Their calculated product defines the density/activity of infected ticks (nymphs and adults/100 m^2^). The error bars depict the upper limit of the 95% confidence interval. Duin&Kruidberg's density of *R. helvetica* infected ticks reaches to 119/100 m^2^.

Identification of high risk-areas depends on both pathogen prevalence and density of questing ticks (nymphs and adults). The density of questing ticks varied between 1/100 m^2^ (Houtvesterijen Heide) and 179/100 m^2^ (Duin&Kruidberg; Table 2). The density of questing *Borrelia*-infected ticks varied between 0 and 19 ticks per 100 m^2^ (Figure 2), whereas the maximum densities of *A. phagocytophilum, N. mikurensis* and *Babesia spp*. infected ticks were 3.0, 13, and 2.9 ticks per 100 m^2^, respectively. The density of questing *R. helvetica*-infected ticks varied between 0 and 22 ticks per 100 m^2^, with one notable exception: Duin&Kruidberg area had both a high tick density and an exceptionally high *R. helvetica* prevalence, which resulted in a density of questing *R. helvetica*-infected ticks of up to 119 ticks per 100 m^2^. Considering that these are calculated as average values for an entire season, it is therefore inevitable that the densities of infected questing ticks are actually higher for peak months of tick activity [i.e., May-June (Gassner et al., 2011)].

**Figure 3:**
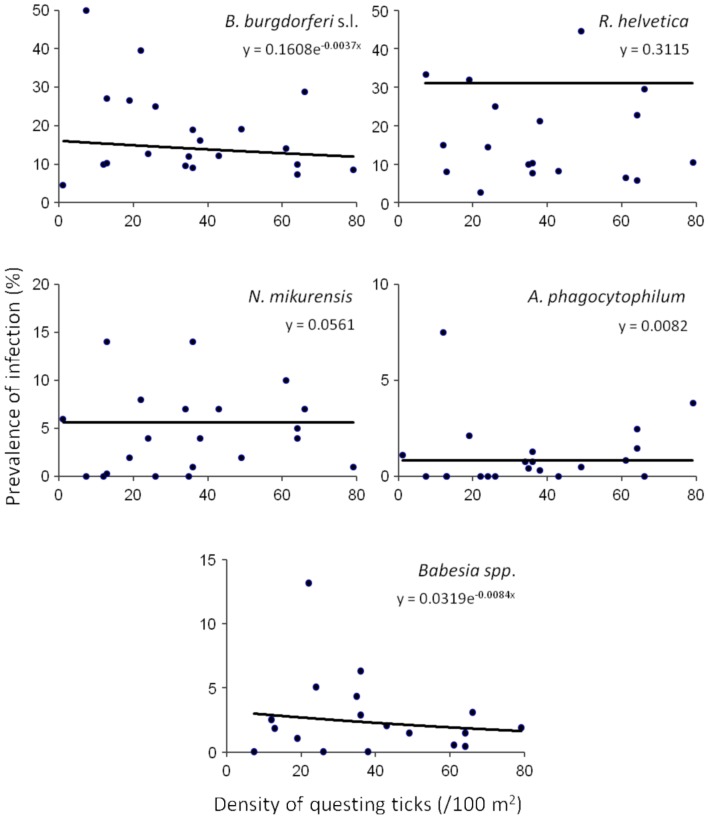
**Density and prevalence relations.** Significant negative correlations between the density of questing ticks and the infection prevalence were found for *B. burgdorferi* s.l. (*p* = 3.6 × 10^−10^) and *Babesia* spp. (*p* = 4.9 × 10^−5^). On the other hand, there was no correlation found between these variables for *R. helvetica* (*p* = 1.0), *N. mikurensis* (*p* = 1.0), and *A. phagocytophilum* (*p* = 0.69). Note that due to the very small exponents, the curves look approximately linear, although they are in fact exponential, as explained in the text. The data set included all of the areas except for Duin&Kruidberg.

**Figure 4:**
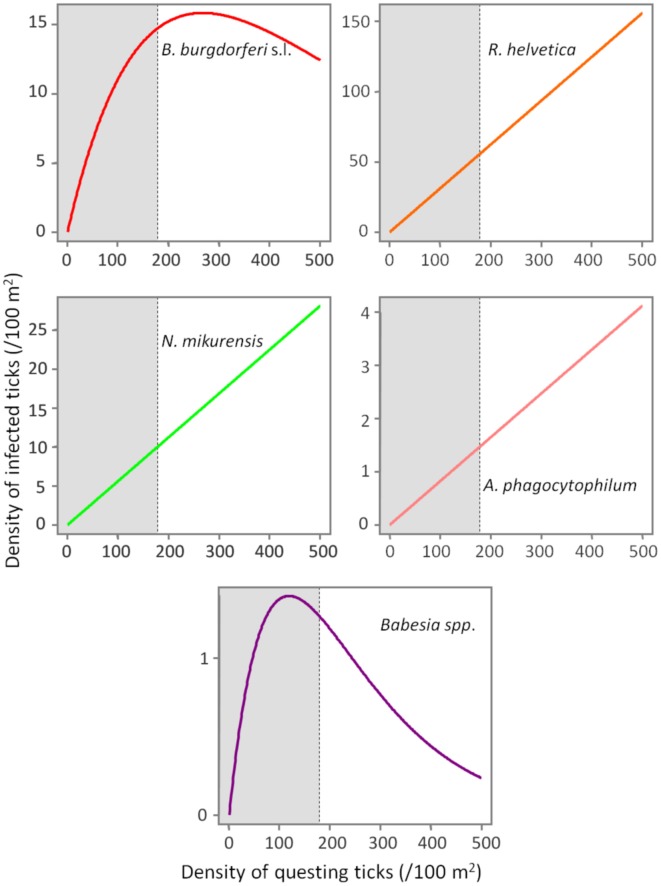
**Evolution of the density of infected ticks (y-axis) with the density of questing ticks (x-axis).** The density of infected ticks is obtained by fitting a model (*p* = *aExp*[*bd*]) to a range of questing ticks densities. The numbers are expressed as ticks/100 m^2^. The gray area marks the normal questing ticks densities (0–179/100 m^2^) in The Netherlands.

Based on a likelihood ratio test, performed for a decreasing model and a constant one, we detected a significant negative correlation between the density of questing ticks and the infection prevalence with *B. burgdorferi* s.l. (*p* = 3.6 × 10^−10^) and *Babesia spp*. (*p* = 4.9 × 10^−5^) (Figure 3). On the other hand, there was no correlation found between these variables for *R. helvetica* (*p* = 1.0), *N. mikurensis* (*p* = 1.0) and *A. phagocytophilum* (*p* = 0.69) (Figure 3). Graphs for the density of infected questing ticks against the density of questing ticks revealed that the former is linearly increasing with the latter for *R. helvetica*, *N. mikurensis* and *A. phagocytophilum* (Figure 4). For the other two pathogens—*Babesia spp*. and *B. burgdorferi* s.l., the density of infected questing ticks reached the maximum values at densities of questing ticks of 119 and 268, respectively (Figure 4).

### Temporal variation

**Figure 5:**
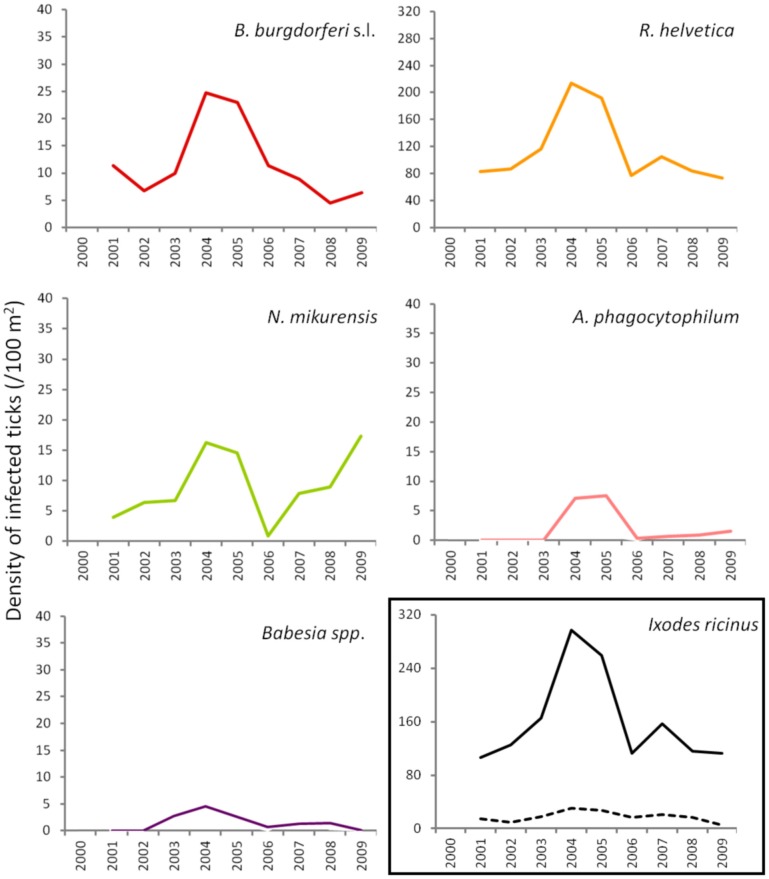
**Changing average (2 years) of density of infected ticks and tick density/activity in Duin&Kruidberg area.** Density/activity of nymphs and adults are shown in the bottom right graph as continuous and dotted line, respectively.

To gain insight into long-term dynamics of ticks and their pathogens, we analysed the data obtained from Duin&Kruidberg, where a 10-year (2000–2009) tick-surveillance was performed. This area was selected at that time because of its unusual high tick density/activity. The prevalences of all pathogens were relatively stable over the past decade (*B. burgdorferi* s.l. 7.0%, *B. afzelii* 4.6%, *A. phagocytophilum* 0.7%, *R. helvetica* 65%, *Babesia spp*. 1.1%), except for *N. mikurensis*, whose prevalence increased from 3.5% (2000–2007) to 12% in the last 2-year interval (2008–2009). The average density/activity of adult ticks remained relatively low with 7–34 ticks per 100 m^2^. The average density/activity of nymphal ticks was more pronounced (102–410 ticks per 100 m^2^) and peaked in 2004–2005 (Figure 5). The likelihood ratio test detected similar decreasing trends in the temporal relation between the prevalence and the tick density as for the spatial variation analysis (not shown). Despite the inverse relationship between the prevalence and the tick density, the peaks of density/activity of *infected* ticks coincided with the peak of high densities of questing ticks in 2004–2005 (Figure 5).

### Coinfection

Overall, 37% (2064/5570) of the ticks was infected with one or more pathogens and 6.3% (350/5570) with more than one pathogen of different genera. Furthermore, 37% (234/628) of the *Borrelia*-positive ticks were infected with at least one other pathogen of a different genus. Almost 5% (29/628) of the *Borrelia*-positive ticks were also positive for three or more other pathogens. One tick carried the DNA of *B.afzelii, R. helvetica, N. mikurensis*, and *B.microti*. Mixed infections, involving two or three *Borrelia* genospecies, occurred in only 0.3% (15/5308) of the tick lysates. Coinfection of *B. afzelii* with *N. mikurensis* or with *Babesia spp*. occurred significantly more than random, whereas infection of *R. helvetica* with either *B.afzelii* or *N. mikurensis* occurred significantly less frequent (Table 3).

**Table 3:** **Observed and expected coinfections**.

	***R. helvetica***	***A. phago-cytophilum***	***N. miku-rensis***	***Babesia spp*.**
**OBSERVED (%)**				
*Borrelia* (all)	3.3	0.1	1.6	0.4
*B. afzelii*	1.8	0.0	1.3	0.3
*R. helvetica*		0.3	2.2	0.5
*A. phagocytophilum*			0.0	0.0
*N. mikurensis*				0.1
**EXPECTED (%)**				
*Borrelia* (all)	3.9	0.1	0.7	0.2
*B. afzelii*	2.2	0.1	0.4	0.1
*R. helvetica*		0.3	1.9	0.5
*A. phagocytophilum*			0.0	0.0
*N. mikurensis*				0.1
**χ^2^-TEST (*p*-VALUE)**				
*Borrelia* (all)	0.03^**^	0.30	0.00^*^	0.01^*^
*B. afzelii*	0.03^**^	0.24	0.00^*^	0.00^*^
*R. helvetica*		0.80	0.05^**^	0.77
*A. phagocytophilum*			0.10	0.42
*N. mikurensis*				0.66

*χ^2^-tests were used to calculate the associations of several combinations of pathogens*.

* Significant positive associations and

** *significant negative associations (*p* < 0.05) are shown in bold*.

### Seasonal dynamics

**Figure 6:**
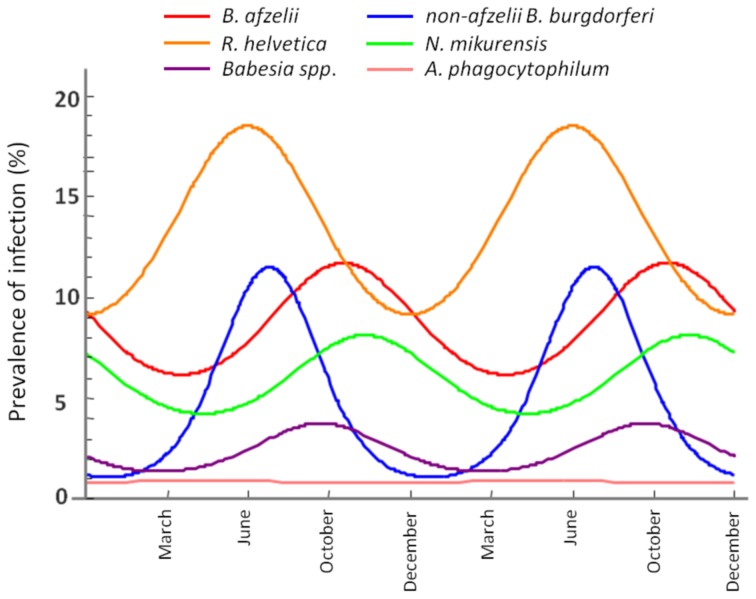
**Seasonal variation of the infection rate in ticks.** The maximum infection rates of non-*afzelii B. burgdorferi*, and *R. helvetica* are in June, while the maximums of *B. afzelii, N. mikurensis*, and *Babesia spp*. overlap in October.

Seasonality modeling of the prevalence indicated a different periodicity of the analysed pathogens (Figure 6). Thus, *B. afzelii, N. mikurensis* and *Babesia spp*. showed highest prevalences in ticks at time periods corresponding to October, while non-*afzelii B. burgdorferi* and *R. helvetica* had the highest prevalence around June. Annual prevalence of *A. phagocytophilum* was not seasonal.

## Discussion

In order to assess the acarological risk of acquiring a tick-borne infection in The Netherlands, the abundance of questing ticks infected with *B. burgdorferi* s.l. and four other genera of TBPs were compared.

Our study revealed the nationwide circulation of TBPs in enzootic cycles. Although the most common tick-borne infection is acknowledged to be Lyme borreliosis, our results showed that there are other pathogens present in questing ticks at prevalences comparable with *B. burgdorferi* (i.e., *R. helvetica*, Table 2). Due to the fact that our investigations only detected the DNA of the microorganisms under discussion, and not the viable cells, we cannot asseverate their infectiousness for other vertebrate hosts. However, previous studies implicate *Ixodes ricinus* ticks as vectors for these microorganisms (Barbour et al., 1983; Ackermann et al., 1984; Lotric-Furlan et al., 1998; Rydkina et al., 1999; Kjemtrup and Conrad, 2000; Parola and Raoult, 2001; Gray et al., 2002; Bonnet et al., 2007; Sprong et al., 2009; Heyman et al., 2010; Jahfari et al., 2012), and therefore the risk for public health should not be neglected. Although no human disease with the organisms other than *B. burgdorferi* s.l. was reported so far in The Netherlands, it is known that infection with some of th0em (e.g., *Ehrlichia*) is generally either asymptomatic or mild, self-limiting diseases (Ismail et al., 2010).

### Spatial distribution and variation

All the pathogens were observed in most of the areas in which investigations were conducted, regardless of the geographical position. The absence in certain areas might be explained by the relatively low number of ticks collected/tested (Table 2). The prevalences of infection in the ticks varied significantly between the areas investigated. The lack of a full perspective on the host community at each of the sites does not allow us to make a definite statement on why we see such a variation of the prevalence of infection. We propose, however, that the extremely high local variability of the pathogens may be associated with the differences in host assemblages in the investigated habitats. As ticks can feed on many different animals and every host species has a unique reservoir competence [e.g., rodents being the most competent reservoirs of *B. afzelii* (Gern and Humair, 2002)], the presence of different hosts in different communities affects the prevalence of infection with various microorganisms.

In terms of the risk for public health, neither the density of questing ticks, nor the prevalence of infection alone, has any significance. Instead, it is their product—the density of infected questing ticks—that defines high or low risk areas (Glass et al., 1994, 1995; Nicholson and Mather, 1996; Dister et al., 1997; Kitron and Kazmierczak, 1997). We noticed that in some areas, where tick densities were highest, the mean prevalence of *Borrelia* infection had very low values (8% for Duin&Kruidberg; Table 2). Using a log-likelihood ratio statistics, we tested the hypothesis of a constant prevalence over the range of questing ticks density. The test confirmed the independence of the two variables but only for *R. helvetica*, *N. mikurensis*, and *A. phagocytophilum*, while for *B. burgdorferi* and *Babesia spp*. it indicated a slight negative correlation of the prevalence with the tick density (Figure 3). Thus, we would expect that the density of ticks infected with *B. burgdorferi* and *Babesia spp*. would decrease as the density of questing ticks increases. Plotting the density of infected questing ticks as an exponential function of the questing ticks' densities, however, revealed that over the usual range of questing ticks densities the density of infected ticks is also increasing, and the downward trend might be observed only for questing ticks densities of over 100 (for *Babesia spp*.) or 200/100 m^2^ (for *B. burgdorferi*) (Figure 4). This observation is consistent with the finding made by Randolph (Randolph, 2001) that, in Europe the density of *Borrelia* infected ticks depends much more on the density of all ticks than on the infection prevalence, and that only in areas where the tick density is unusually high (100–450/100 m^2^) is the infection prevalence consistently low.

### Temporal variation

In terms of temporal variation, the longest series of data we had was for 10 successive years (Duin&Kruidberg, Figure 5). At this site, the density of questing ticks was highest in 2004–2005, and it was due to a steep increase in the number of questing nymphs. The variations in tick density might indicate yearly fluctuations in the composition and availability of reservoir hosts. For example, a mast year might have been responsible for the increment in small mammals' population size (i.e., rodents), with the upsurge of nymphs at a consequential rate. The trend line indicated the maintenance of relatively constant prevalences for *B. burgdorferi, A. phagocytophilum*, and *R. helvetica.Babesia* prevalence showed a slight decrease over time while, on the contrary, *N. mikurensis* showed a steep increase (almost 3-fold). The maintenance of relatively constant prevalences of infection in time implies that the acarological risk is predominantly dependent on the density/activity of ticks (Figure 5).

### Coinfection

One-third of the ticks infected with *Borrelia* were also infected with at least one other TBP. Recent studies in other European countries have shown that mixed infections of the TBPs do not represent an exception but more likely the rule.

A negative significant association was found between all *Borrelia* (and *B. afzelii* alone) and *R. helvetica*, as well as between *N. mikurensis* and *R. helvetica* (Table 3). On the other hand, significant positive associations were found between *Borrelia* (and particularly *B. afzelii*) and *N. mikurensis* and between *Borrelia* and *Babesia spp*. (Table 3). These findings lead us to the hypothesis that *B. afzelii, N. mikurensis*, and *Babesia* might share the same reservoir hosts, while *R. helvetica* is maintained in other enzootic cycles.

### Seasonal dynamics

Further evidence for our hypothesis came from the seasonality modeling of the infection prevalence. This indicated a variation in the same phase for *B. afzelii, N. mikurensis* and *Babesia spp*. on the one side and for non-*afzelii B. burgdorferi* and *R. helvetica* on another (Figure 6). That means that the infection peak in questing ticks is different for different pathogens, further suggesting that they were acquired from the distinct vertebrate hosts. Scientific literature confirms this. Rodents are known to be competent transmission hosts for *B. afzelii* (Gern and Humair, 2002; Hanincova et al., 2003a) and *B. microti* (Gray et al., 2002), and they have been designated as potential reservoirs for *N. mikurensis* (Ginsberg, 2008; Andersson and Raberg, 2011). On the other hand, non-*afzelii Borrelia*, like *B. garinii* and *B. valaisiana* have been shown to be associated with birds (Gern and Humair, 2002; Hanincova et al., 2003b), while a study of de la Fuente and co-workers (De La Fuente et al., 2005) found that *A. phagocytophilum* infections occurred in deer, cattle and various bird species, meaning that birds might serve as reservoirs for both these bacteria. *R. helvetica* was previously found at high rates in both rodents (29%) and roe deer (19%) (Sprong et al., 2009). The fact that *R. helvetica* was negatively associated with *B. afzelii*, although they might share the same hosts, is possibly due to that the former is transovarially transmitted in ticks which act thus as both vectors and reservoirs of the rickettsiae (Sprong et al., 2009); therefore, they alone can be responsible for the maintenance of the bacteria, without the intervention of a rodent host in the cycle. Hence, our findings are not coincidental, and indicate that certain coinfections are more likely to occur than the others, given particular combinations of vertebrate hosts.

Although previous meta-analyses indicate that coinfection and co-exposure for some of the TBPs appear to occur somewhat unpredictably across different areas and different hosts (Nieto and Foley, 2009), it is anticipated that future wildlife studies will help define geographical risks of coinfection and provide insight into the dynamics of infection within reservoir hosts.

## Conclusion

We have shown that ticks and the five genera of TBPs have a ubiquitous distribution in The Netherlands, with the few absence point presumably determined by the small number of collected ticks. The pathogens were found in sites all over The Netherlands, encompassing a variety of habitats, from open areas such as dune and heather to deciduous or coniferous forests.

This study brings valuable information on the prevalence, geographic distribution and temporal variation of *B. burgdorferi* s.l., *R. helvetica, N. mikurensis, A. phagocytophilum* and *Babesia spp*. in questing *I. ricinus*. Due to their omnipresence, we underline the need to consider all of these pathogens when evaluating the risks of infection and subsequently of disease following a tick bite.

Whereas the incidence of Lyme disease is on the rise, other tick-borne diseases remain heavily unreported, and even knowledge on the human exposure to them is scarce. Our study suggests that there are pathogens positively associated with *Borrelia* (i.e., *N. mikurensis* and *Babesia spp*.) in questing ticks. This strengthens the idea of established enzootic cycles (common reservoir hosts) in which these microorganisms are maintained, and it is consequently possible that they might follow the same upward trend as the Lyme spirochetes. In the case of *N. mikurensis* we have in fact witnessed the beginning of what might be a following upward trend.

Human activity in any natural habitat is accordingly accompanied by an imminent risk of exposure to any of the pathogens. Although the risk, as measured by the density of infected ticks, may vary in time and space, its driving factor appears to be the tick density/activity. It is therefore possible that the risk of exposure to TBPs would be minimized by developing effective and sustainable methods for the control of *Ixodes ricinus* populations.

## Author contributions

Hein Sprong, Willem Takken, and Joke van der Giessen designed parts of the study. Catharina B. Maassen and Willem Takken organized the collection of ticks and field data. Manoj Fonville performed lab tests and laboratory analyses. Elena Claudia Coipan and Setareh Jahfari analysed data and performed statistical analysis. Katsuhisa Takumi performed mathematical analyses. Hein Sprong, Joke van der Giessen, and Willem Takken supervised different parts of study. Elena Claudia Coipan and Hein Sprong wrote the final manuscript. All authors read and approved the final manuscript.

### Conflict of interest statement

The authors declare that the research was conducted in the absence of any commercial or financial relationships that could be construed as a potential conflict of interest.
